# HIV Infection and Related Mental Disorders

**DOI:** 10.3390/brainsci11020248

**Published:** 2021-02-17

**Authors:** Marina Nosik, Vyacheslav Lavrov, Oxana Svitich

**Affiliations:** I.I. Mechnikov Research Institute of Vaccines and Sera, 105064 Moscow, Russia; v.f.lavrov@inbox.ru (V.L.); svitichoa@yandex.ru (O.S.)

**Keywords:** HIV-1, CNS, CNS cells, neurocognitive disorders (HAND), ART, psychotropic drugs, drug–drug interactions

## Abstract

Over the more than thirty-year period of the human immunodeficiency virus type 1 (HIV-1) epidemic, many data have been accumulated indicating that HIV infection predisposes one to the development of mental pathologies. It has been proven that cognitive disorders in HIV-positive individuals are the result of the direct exposure of the virus to central nervous system (CNS) cells. The use of antiretroviral therapy has significantly reduced the number of cases of mental disorders among people infected with HIV. However, the incidence of moderate to mild cognitive impairment at all stages of HIV infection is still quite high. This review describes the most common forms of mental pathology that occur in people living with HIV and presents the current concepts on the possible pathogenetic mechanisms of the influence of human immunodeficiency virus (HIV-1) and its viral proteins on the cells of the CNS and the CNS’s functions. This review also provides the current state of knowledge on the impact of the antiretroviral therapy on the development of mental pathologies in people living with HIV, as well as current knowledge on the interactions between antiretroviral and psychotropic drugs that occur under their simultaneous administration.

## 1. Introduction

HIV-1 (human immunodeficiency virus type 1) is one of the most dangerous and widespread infectious viruses and causes the deaths of millions of people. The global spread of this virus, which has taken on the character of a pandemic, has made HIV a central health problem worldwide. Today, there are about 38 million people living with an HIV infection globally [1]. In 2019 alone, 690,000 people died from HIV-related causes, and 33 million have died since the beginning of the epidemic [1]. Currently, thanks to the active development of innovative forms of antiretroviral drugs and increased access to effective means of prevention, HIV infection has become a non-fatal, and in many cases, chronic disease. Thus, the life expectancy of people living with HIV (PLWH) has significantly increased [2,3]. At the same time, in the population of PLWH, in addition to the consistently observed higher rates of morbidity and mortality from cardiovascular diseases, various metabolic complications, and non-AIDS-related malignancies [4], there is a clear trend towards the spread of neurocognitive disorders [5,6,7,8,9], which reduce the quality of life among these people. It has been shown that HIV-positive patients are 2–4 times more likely to develop depression than HIV-negative individuals [10]. At the same time, even a mild form of depression is a serious factor that worsens the health of PLWH, starting with significant dysregulation of the immune response and ending with an increase in mortality rates [10]. Clearly, the mental disorders associated with the infection of people with human immunodeficiency virus type 1 (HIV-1) are a serious problem faced by physicians in the treatment of PLWH. In this regard, it is extremely important to determine the full range of mental disorders observed in HIV infection, as well as the factors that affect the occurrence and development of mental pathologies in HIV-positive patients. When choosing a treatment regimen, the results of the interactions between simultaneously prescribed psychotropic and antiretroviral drugs that occur in patients should be considered. This review describes the most common forms of mental pathology associated with HIV infection, presents the current concepts on the possible pathogenetic mechanisms of the influence of HIV and its viral proteins on the central nervous system (CNS) and its functions, summarizes current research on the impact of antiretroviral therapy on the development of mental pathologies, and outlines the interactions between widely used antiretroviral drugs and psychotropic drugs.

## 2. Mental Disorders Associated with HIV Infection

The relationship between HIV infection and mental illness is rather complex and largely unexplored to date. It has been shown that people with severe mental disorders have a significantly increased risk of HIV infection [11,12]. It was also found that the percentage of HIV infection in patients with a mental pathology is, on average, seven times higher than that among mentally healthy people [11,12]. It is believed that this is due to the distortion of the processes of perception and thinking in persons suffering from mental disorders, their use of psychoactive substances, their risky sexual behavior, and sexual victimization [11,12]. Thus, it has been found that an increased risk of HIV infection is directly associated with hypersexuality during an exacerbation of mental illness. It was noted that the frequency of sexual activity in the acute phase of schizophrenia increased in 38.6% of individuals and in 44.8% of individuals with bipolar disorders, the frequency of sexual activity in individuals with mental illnesses under the influence of heroin increased in 43.4% [13]. Such patients had sexual behavior associated with an increased risk of sexual transmission of HIV: 39–42.7% had intercourse with several sexual partners at the same time; 24% had sex with prostitutes, and in doing so, 65% had unprotected sexual intercourse (of which 12.5% had unprotected sexual intercourse in order to earn money), [13,14]. Additionally, as a result of impaired cognitive abilities, evaluation, and judgment, people with mental disorders are much more likely to be at risk of coerced sex [13,14,15,16,17,18]. The frequency of forced sexual intercourse among this group of individuals is 10–38% [14,19,20]. Many patients with mental illnesses such as schizophrenia and bipolar disorder have been found to have experienced sexual abuse in childhood [17,19,21]. The traumatic experience is later reproduced in life and such persons repeatedly become victims of sexual violence; they are characterized by sexually promiscuous and risky sexual behavior in adulthood [17,22].

In turn, over the more than thirty years of the HIV epidemic, many materials have been accumulated indicating that HIV infection predisposes one to the development of a mental pathology such as anxiety disorders, bipolar disorders, schizophrenia, and cognitive disorders. It has been observed that the early stages of HIV infection are most often accompanied by a depressive state [10,23]. The progression of HIV infection is then characterized by the development of psychosis, adjustment disorder, and bipolar disorder [11,24,25]. In the African continent, where the burden of HIV infection is particularly high, the prevalence of HIV infection among adults with severe mental disorders ranges from 11 to 48.6% [26,27,28,29,30,31]. The results of a multisite study conducted in the United States showed that 36% of people living with HIV (PLWH) suffered from severe depression, 15.8% suffered from generalized anxiety disorder (GAD), and 10.5% suffered from panic disorder (PD)—three times higher than similar indicators among the general population [32,33,34]. At the same time, a combination of GAD and PD was diagnosed in 5% of PLWH [33]. Thus, it can be stated that HIV infection is a serious predictor of the development of severe forms of depression, GAD, and PD [35]. It should be noted that early initiation of antiretroviral therapy (ART) does not reduce the risk of any of these mental disorders. In India, the number of HIV-infected people with severe forms of depression is 59%, and in China, this number ranges from 32.9 to 85.6% [10,36,37,38]. It is found that depressive conditions in PLWH significantly increase the risk of death [39,40]. An analysis of the case histories of 5927 HIV-infected people showed that mild and short-term (1–4 days) depression and/or mild but more persistent depressive conditions can negatively affect the process of HIV treatment and the survival of PLWH [23].

It was revealed that psychosis is more often diagnosed in patients with severe immunodeficiency (CD4 ≤ 200–350 cells/mm^3^), which usually occurs in the late stages of HIV infection. The frequency of the first psychotic episodes in HIV-positive individuals ranges from 1 to 15% [41,42]. For psychoses arising in HIV-infected patients, hallucinations, affective disorders, cognitive impairments, and dementia are common [43,44]. Moreover, the risk of schizophrenia and acute psychosis in people infected with HIV during the first year after infection is quite high, with an incidence rate of 8.24 and 12.7, respectively [41]. Moreover, an increased risk of developing schizophrenia persists for more than 5 years after the diagnosis of an HIV infection, at which point the majority of PLWH receive antiretroviral therapy (ART), leading to suppression of viral replication and of opportunistic infections [41]. It was found that the comorbidity of HIV infection and schizophrenia among such patients significantly correlates with a high risk of lethal cases [25,41]. The results of two large-scale studies showed that among patients with schizophrenia, there was a higher mortality rate in the group of HIV-positive individuals compared to the group of HIV-negative patients [25,41]. A six-year follow-up of such patients revealed that the mortality among HIV-positive patients diagnosed with schizophrenia was 25.5%, while that among HIV-negative patients with the same diagnosis was only 17.8% [25]. Another more long-term (12-year) study found that the mortality rate in the group of patients with HIV and schizophrenia was 25.8, while in those with schizophrenia but not infected with HIV, this indicator was 6.24 [41].

According to the materials of the short international neuropsychiatric questionnaire (MINI), cases of bipolar disorder among PLWH in economically developed countries are 5.6–8.1%, which is 3–4 times higher than the same indicators among the general populations of these countries (2.1%) [45,46,47]. In developing countries, this figure reaches 30% [48]. Manic disorder in HIV-positive individuals during later stages of the infection often occurs as a phase of bipolar disorder and is likely associated with the direct effect of HIV on the cells of the central nervous system (CNS). Manic disorder can also result from a secondary infection or be influenced by ART. It was found that severe cognitive impairment occurred in 54.8% of people with HIV-associated mania, while in HIV-negative people with the initial stage of that same mental pathology, such disorders occurred in only 15.9% [38]. It was also shown that HIV-associated manic disorder represents the initial stage of HIV-associated dementia [49,50,51], which occurs in 10% of HIV-positive individuals during the late stages of HIV infection [52].

## 3. Biological Mechanisms of HIV-1—Effect on CNS Functions

It has now been proven that neurocognitive changes in HIV-positive individuals are the result of the direct effect of HIV on the central nervous system (CNS). It was found that following the first days after infection with human immunodeficiency virus type 1 (HIV-1), this viral agent penetrates into the tissues of the CNS [53] and localizes in the various parts of the brain [54,55]. Viral RNA was found in the caudate nucleus, the cortex of the frontal lobe of the brain, and the cerebrospinal fluid. HIV-1 localized in the basal ganglia of the CNS causes progressive neurodegenerative changes, as well as impaired neuromotor and neurocognitive functions. A number of studies have shown that the development of HIV infection is accompanied by a decrease in the volume of the caudate nucleus and white matter, as well as the cortical and subcortical gray matter of the brain [56,57,58], which is directly related to bipolar disorders [59]. Dysregulation in the metabolic activity of the basal ganglia was also identified. These phenomena were shown to be expressed in the form of hypermetabolism in the early stages of HIV infection and hypometabolism in the later stages of the infectious process [60].

It is believed that HIV-1 enters the CNS via the migration of virus-infected mononuclear blood cells/monocytes using the “Trojan horse mechanism”, which allows the virus to cross the blood–brain barrier (BBB) and infect the astrocytes, oligodendrocytes, and progenitor cells [5,61,62]. It has been shown that HIV-1 can change BBB permeability by modifying the expression of proteins involved in the maintenance of a dense barrier epithelium through the action of the viral *tat* protein [63,64]. Free viral particles can also penetrate the BBB via transcytosis, which is mediated by the viral protein *gp120* [65] or by productive infection of the endothelial cells [66,67]. Some researchers believe that the freely circulating proteins *gp120*, *tat*, and *nef* can bind to the microvascular endothelial cells and cause changes in the BBB without direct involvement of the virus [68]. However, the main targets of HIV-1 invasion are the macrophages and microglia cells upon whose surfaces the co-receptors CXCR4 and CCR5 are expressed, which are necessary for the virus to enter cells [69,70,71] and which become cellular reservoirs for the long-term persistence of the virus in the CNS, thus playing an important role in the development of HIV-induced dementia [61,72,73]. The cerebrospinal fluid can also serve as a reservoir for the virus, which was confirmed by the results of the studies showing a high level of HIV-1 RNA in the cerebrosinal fluid of AIDS patients [74,75].

By penetrating into the CNS, HIV-1 induces an increase in the expression of chemokine receptors, the production of inflammatory mediators, the production of enzymes that destroy the extracellular matrix, and excitotoxicity mediated by glutamate receptors, which, in turn, initiates the activation of numerous downstream signaling pathways and disrupts neuronal and glial functions [61,76].

It has been shown that the viral protein *tat* can cause activation of the effector pyrine domain of the Nod-like receptor (NLR) containing the NLRP3 of the inflammasome in microglia, which leads to an increase in the levels of caspase-1 and IL-1β; this, in turn, induces the production of TNF-α and IL-6, thereby enhancing inflammatory processes [77]. In addition, HIV-1 infection of the microglial cells and macrophages induces the production of reactive oxygen species (ROS) and reactive nitrogen species (RNS), which disrupts the functions of the signaling pathways associated with apoptosis and leads to cell cycle arrest, causing serious DNA damage and protein damage [78,79].

In astrocytes, in contrast to microglia and macrophages, the full replication of HIV-1 is limited [80,81]. These cells cannot produce full-fledged viral particles, but, at the same time, they contribute to the damage of brain cells by generating astrogliosis [82]. Virus-infected astrocytes can produce a number of viral regulatory proteins, such as *tat*, *nef*, and *rev*, which are involved in the development of inflammation and, therefore, neuronal damage [83]. It is known that *tat* activates HIV-1 transcription and enhances the process of the infection of primary astrocytes [84]. On the other hand, *nef* can induce the production of ROS by astrocytes, which leads to the rapid death of neurons, thereby causing the development of HIV-1-associated neurocognitive disorders and explaining the reason for the rapid development of dementia in patients not receiving ART or with low adherence to treatment [85].

Despite the fact that some oligodendrocytes express the CXCR4 co-receptor (one of the key receptors involved in the infection of HIV-1 cells) [86], it is believed that the most profound damage to oligodendrocytes is caused by the release of viral proteins from other cells infected with the virus [87]. It has been found that the viral *tat* protein promotes the death of oligodendrocytes or leads to their incomplete maturation, as well as the dysregulation of myelin protein expression, which reduces the ability of oligodendrocytes to create myelin sheaths [88,89]. *Tat-*induced damage to oligodendrocytes is associated with a change in the balance between the protein kinase CaMKIIβ and tyrosine kinase 3β (Gsk3β), leading to oligodendrocyte apoptosis and the development of a neuropathology [89].

Neurons cannot be infected with HIV-1, because they do not express the virus-specific receptors necessary for the virus to enter these cells. However, the viral proteins *tat*, *nef*, and *gp120*, which have high neurotoxic potential, can disrupt interneuronal connections and even cause neuronal death [90]. The *tat* protein interacting with the markers of the phagosomes in neurons changes the morphology of these formations, thereby preventing their fusion with lysosomes [91]. The *nef* protein has a similar effect, disrupting the autophagy process and causing neurodegenerative disorders [92].

Table 1 summarizes the pathological effect caused by the HIV-1 on CNS cells.

## 4. Impact of Antiretroviral Therapy (ART) on the Development of Mental Pathologies

There is no doubt that ART has significantly reduced the incidence of HIV-related mental disorders. Severe cognitive impairments in HIV-infected patients taking antiretroviral drugs are rarely diagnosed and are observed now in only 2% of cases [6,8,93]. However, despite a decrease in the viral load to an undetectable level (less than 40 copies of RNA copies/mL) and the restoration of normal immune status in patients, the incidence of moderate and mild cognitive impairments at all stages of HIV infection still remains rather high, with 12–52% moderate and 33% asymptomatic cognitive impairments [5,6,7,94,95]. According to the experts, this may be due to a number of reasons, including the difficulty in overcoming the BBB with antiretroviral drugs. Some of these drugs cannot completely penetrate the CNS tissues and, therefore, effectively suppress viral replication [5,6,96]. This was confirmed by the results of a quantitative PCR analysis, which detected HIV-1 RNA in most tissue samples taken from the different parts of the brain during an autopsy of HIV-infected patients who received ART [54]. In another study, abnormal levels of microglial activation were found in HIV-infected individuals with asymptomatic cognitive impairment and a low viral load [97]. It is possible that even relatively low replicative activity of the virus in the CNS can cause damage or dysfunction of the neurons, which is likely a consequence of the neurotoxic effect of the viral proteins or the result of a prolonged immune impact in response to the products of microbial translocation [5,6,98]. In addition, there is an assumption that in people with long-term HIV infection, the risk of metabolic disorders and associated pathological changes in the brain vessels will increase [5,80]. Moreover, the negative effects of β-amyloid deposits in the brain tissues cannot be ruled out [5,80].

Recent publications indicated that antiretroviral drugs themselves can cause neurotoxic effects and contribute to the development of cognitive impairment. It was noted that in patients who stopped treatment, cognitive functions improved significantly [7]. At the same time, in patients with a low viral load who continued treatment, there was a deterioration in the indicators of neurological status [99]. It was also found in vitro (on the model of primary rat cortical neuroglial cultures) that co-administration of the antiretroviral drugs according to a scheme of using one nucleoside analog + two drugs of the class of protease inhibitors (PIs)—1 NRTI + 2 PIs—causes a neurotoxic effect even in HIV-uninfected cultures [100]. One of the main markers of brain activity is N-acetylaspartate (NAA), an amino acid, localized in neurons and gray matter and synthesized by brain mitochondria from acetyl coenzyme A and aspartate, which uses the enzyme aspartate N-acetyltransferase [101]. Mitochondria play an important role in the proper functioning of nerve cells, and their dysfunction is the basis for the development of numerous neurodegenerative diseases. It was found that fluctuations in the NAA level are a reliable indicator of impaired mitochondrial metabolism [101]. It has been shown that a number of antiretroviral drugs of the NRTI class (nucleoside reverse transcriptase inhibitors) have mitochondrial toxicity [102,103,104,105,106]. It has been noted that PLWH taking stavudine and/or didanosine experience a 11.4% decrease in the concentration of NAA in the white matter of the frontal lobe of the brain compared to HIV-negative individuals and PLWH receiving a different ART regimen [102]. Long-term use of these drugs can lead to a decrease in the concentration of NAA. Taking several drugs of the NRTI class at once (stavudine, didanosine, and/or abacavir) significantly increases the likelihood of a decrease in the NAA level [102]. It is assumed that a decrease in NAA concentration is most likely a consequence of the depletion of brain mitochondria and/or impaired cellular respiration. Drugs of the NRTI class are inhibitors of mitochondrial DNA polymerase γ (Pol-γ), which is responsible for the mitochondrial DNA (mtDNA) replication [103,105,107]. It has been suggested that prolonged inhibition of pol-γ induced by NRTIs leads to impaired mtDNA synthesis, followed by mtDNA depletion, disruption of the respiratory electron transport chain (ETC), disruption of oxidative phosphorylation (OxPhos), and a decrease in the ATP pool [103,104,106]. Since NRTIs and endogenous nucleosides share the same nucleoside transport and phosphorylating systems, it is assumed that mitochondrial toxicity induced by NRTIs may arise due to their competition with natural nucleoside/nucleotide pools, which leads to mtDNA depletion [104,108]. As the synthesis of pyrimidine nucleotides by dihydroorotate dehydrogenase is directly related to the functional ETC, it is possible that these disorders may be secondary regarding the impairment of the respiratory electron transport chain [104,109]. Disruption of ETC and OxPhos processes may further enhance NRTI-associated mitochondrial toxicity, leading to a constant accumulation of toxic effects [104]. In addition, as a result of impaired mitochondrial function and impaired electron transport, there is an increase in the production of reactive oxygen species (ROS) in the mitochondrial matrix, which in turn can increase the frequency of mtDNA mutations and, as a consequence, lead to the enhanced mitochondrial dysfunction [103,104,110,111]. Relatively recently, another hypothesis of mitochondrial toxicity caused by NRTIs, independent of pol-γ inhibition, has emerged, according to which drugs of this class disrupt endogenous ribonucleotides (RNs) and deoxyribonucleotides’ (dRNs) pools, leading to their depletion [112]. Currently, the mechanism by which nucleoside analogs are transported to the mitochondrial matrix is still unclear. However, since nucleotides and their analogues are hydrophobic molecules, it is believed that they require nucleotide transport proteins, in particular, concentrative nucleoside transporters (CNTs) and equilibrative nucleoside transporters (ENTs), to penetrate through cell membranes and enter the mitochondrial matrix [103,112,113]. It is assumed that, as a result, RNs and dRNs’ pools may be affected by nucleotide transport proteins and adenosine triphosphate-binding cassette (ABC) proteins [112,114].

It has been proven that a widely used drug of the NNRTI class (non-nucleoside reverse transcriptase inhibitor), efavirenz (EFV), is capable of causing neuropsychiatric side effects, such as impaired concentration, anxiety, nightmares, and hallucinations [115,116,117]. A nine-year study involving 5332 participants (*n* = 3241 receiving EFV and *n* = 2091 not receiving EFV) showed that patients treated with an ART regimen containing efavirenz had a twofold increase in suicidal ideation compared to the patients on an ART regimen without efavirenz [118]. Another 4-year large-scale study involving 4684 participants from 35 countries (*n* = 3515 (75%) received EFV) also confirmed an increased risk of suicidal behavior in patients taking EFV compared to ART-naive patients [119]. Here, individuals with a previous history of psychiatric diagnoses were at a higher risk of developing suicidal ideation [119]. The mechanisms responsible for the neurotoxic effect associated with the use of efavirenz are not fully understood. In vitro studies using a primary culture of rat cortical neurons showed that EFV causes disfunction in the functional activity of the mitochondria, including a decrease in ATP production, as well as mitochondrial fragmentation and depolarization [120]. Another in vitro study (on the model of human glioma cell line and human neuroblastoma cell line) showed that EFV administration inhibits mitochondrial membrane potential and mitochondrial respiration and induces increased ROS generation in both neurons and glia, but a difference in ATP expression was found [115]. The drug induced an enhancement in 5′ AMP-activated protein kinase (AMPK) in the glioma cell line, which led to the upregulated glycolysis and high levels of ATP production [115]. In neurons, EFV induced a decrease in ATP [115]. This suggests that EFV affects the energy balance of CNS cells through a mechanism involving mitochondrial inhibition.

## 5. Competitive Interaction of Psychotropic and Antiretroviral Drugs in the Treatment of Mental Pathologies in HIV-Infected Patients

Many antiretroviral drugs, as well as antidepressants and neuroleptics, are metabolized by the cytochrome P450 isoenzymes. When co-administered together, they are often subjected to competitive inhibition, which leads to an acceleration of their metabolism, a decrease in their concentration in the blood, and a weakening of their clinical effect [121]. Conversely, the combined use of psychotropic and antiretroviral drugs can cause a decrease in the metabolism of both; as a result, the concentration of these drugs in the blood increases, and patients develop extrapyramidal symptoms (EPS) [122]. Table 2 lists drug-drug interactions between these classes of medications.

It has been shown that drugs such as *fluoxetine and paroxetine*, which are selective serotonin reuptake inhibitors (SSRIs), prescribed for severe forms of depression and obsessive-compulsive disorder, are strong-acting inhibitors of cytochrome CYP2D6 (an enzyme responsible for the metabolism and elimination of approximately 25% of clinically used drugs). Moreover, when administered concomitantly with antiretroviral drugs belonging to the protease inhibitors class (PI), SSRIs can cause a toxic effect due to the increased concentration of PIs [121,122]. With co-administration of ritonavir (PI) and fluoxetine, a 19% increase in the area under the curve (AUC) of ritonavir was observed, causing cardiovascular and neurological complications [122,123,124]. When fluoxetine was co-administered with a drug of the NNRTI class, delavirdine, the concentration (Cmin) of the latter can increase by 50%, although the manufacturer does not recommend any changes in the dose of the drug [125]. In contrast, the combined use of fluoxetine with the NNRTI class drug nevirapine can reduce the level of fluoxetine in blood plasma by almost two times [126]. It was also noted that the combined use of the antidepressants paroxetine or sertraline with ritonavir/darunavir (PI class drugs) can reduce the concentration of paroxetine, on average, by 37–40% [127,128] and sertraline by 47% [128]. Co-prescribing the NNRTI-class drugs efavirenz and sertraline also leads to a concentration decrease in the latter (by 39%) [128]. In this regard, the recommended approach in combination treatment is to carefully select the dose of an antidepressant, taking into the account the clinical assessment of the response to this drug. In addition, in patients receiving stable doses of antidepressants and prescribed ritonavir/darunavir combination therapy, the clinical response to the antidepressants’ actions should be monitored [128]. Fluvoxamine, which is prescribed for severe depression and is a strong inhibitor of CYP1A2 (an enzyme involved in the metabolism of xenobiotics in the body) can also cause a toxic effect resulting from an increase in the concentration of PI drugs in the blood [121]. However, when fluvoxamine is co-administered with nevirapine (NNRTI), nevirapine clearance decreases by 33.7% [126].

Trazodone prescribed to patients with severe forms of depression is metabolized by CYP3A4 (an enzyme involved in the metabolism of xenobiotics) and CYP2D6, so its combined administration with the antiretroviral drugs of the PI class that inhibit these enzymes may cause an increase in the concentration of trazodone in the blood of patients [121,122]. It was shown that the short-term use of ritonavir (PI) resulted in a significant prolongation of the half-life of trazodone (the average clearance was less than 50% of the control values), which caused nausea, dizziness, and psychomotor disorders in the study participants [129]. Increasing the therapeutic dose of trazodone may cause cognitive impairment, hallucinations, confusion, and suicidal ideation [130]. Therefore, in the initial stages of treatment, it is recommended to prescribe trazodone at a minimum dose, with careful monitoring of its side effects [128].

In vitro studies have shown that the combined use of nelfinavir (PI) or efavirenz (NNRTI), which inhibit CYP2B6 (an enzyme involved in drug metabolism, cholesterol synthesis, and steroids) and bupropion (a monocyclic antidepressant metabolized by CYP2B6), increases the concentration of this antidepressant in the blood [131]. However, an in vivo study involving healthy volunteers showed that the combined use of efavirenz with bupropion leads to a decrease in the level of bupropion in the blood by an average of 55% [132,133]. It has also been shown that the combined use of bupropion and ritonavir (PI), which can both inhibit and induce the expression of CYP2B6, leads to a decrease in the concentration of the antidepressant, depending on the dose of ritonavir [122,134]. Thus, high doses of ritonavir (600 mg) reduced the area under the curve (AUC) of bupropion by an average of 65% and decreased the time to reach the maximum concentration (Tmax) by 62%, while low doses of this drug (100 mg) reduced these indicators by 22 and 21%, respectively [134].

The use of ritonavir (PI) together with tricyclic antidepressants (TCAs), which are also metabolized by CYP2B6, can lead to a 1.5–3-fold increase in the concentration of TCAs, which increases the toxic effects of these antidepressants, causing agitation, confusion, and delirium in patients [135]. The toxic effects of TCAs can be accompanied by cardiac arrhythmia, convulsions, and—in some cases—lead to death [135]. In this regard, the treatment of HIV-positive patients with TCAs (amitriptyline, desipramine, doxepin, imipramine, and nortriptyline) should be started with low doses of the drug followed by carefully increasing the dose, if necessary, based on the therapeutic effect and the concentration of the drug in the blood [128].

The neuroleptics risperidone and aripiprazole, which are prescribed for the treatment of schizophrenia and acute mania, are metabolized by CYP2D6 and CYP3A4 isoenzymes, so their combined use with PI drugs such as indinavir (a CYP3A4 inhibitor), ritonavir, and danuravir (CYP3A4 and CYP2D6 inhibitors) can lead to an increase in their concentration in the blood and the development of various side effects, including dizziness and confusion [121,122,136]. Thus, the combined administration of ritonavir/danuravir and aripiprazole increased the concentration of the neuroleptic by five times compared to the therapeutic dose [136]. In this regard, it is recommended to prescribe 25% of the usual dose of aripiprazole and, if necessary, increase the dose of the antipsychotic based on the clinical monitoring of adverse events [128]. Under the co-administration of the neuroleptics quetiapine and ziprasidone (which are metabolized by CYP3A4) and strong CYP3A4 inhibitors, such as ritonavir and other PI drugs, an increase in the concentration of antipsychotics in the blood can be observed [122,137]. In patients, this may cause panic attacks, insomnia, irritability, hostility, aggressiveness, impulsivity, hypomania, and/or mania, which may be precursors to suicidality [137]. In this regard, the combined use of darunavir/ritonavir with quetiapine is contraindicated due to the possible increase in quetiapine-related toxicity and the occurrence of a comatose state [128]. Conversely, the simultaneous administration of olanzipine with ritonavir (PI) leads to a decrease in the concentration of this neuroleptic, since ritonavir accelerates its half-life in the body [135,138]. At the same time, the clearance rate depends on the PI dose: At a 500 mg dose of ritonavir, the AUC of olanzapine decreases by 53% [135], and at a 100 mg dose of ritonavir (as part of the combined drug with 700 mg fosamprenavir and 100 mg ritonavir), the half-life of olanzapine decreases by an average of 32% [138]. Therefore, when co-administrating ritonavir and olanzapine, it is recommended to increase the dose of the latter by 50% [138].

Anxiolytics such as benzodiazepines (alprazolam, midazolam, triazolam, and estazolam), which are prescribed for panic attacks, anxiety disorders, and sleep disorders, are metabolized by CYP3A4, so their co-administration with ritonavir (PI), which is a CYP3A4 inhibitor, can reduce the clearance of benzodiazepines and lead to an overdose [121,133]. It has been shown that the combined use of alprazolam with ritonavir reduces the clearance of anxiolytics by 41%, which can cause side effects such as difficulties in coordination, irritability, and problems with concentration, routine tasks, sleep, and speech [139,140]. Administration of the PI class drug saquinavir together with midazolam reduces the half-life of the drug and increases its concentration (Cmax) in plasma by 2–3 times, thus increasing the bioavailability of the drug from 41 to 90% [141,142]. It has been shown that co-therapy with the anti-retroviral drugs of the NNRTI class, etravirine and midazolam, increases the effect of the latter, enhancing the concentration (Cmax) of the drug in the blood by 57% [128]. An overdose of midazolam can lead to cardiac depression, impaired coordination of movements, paradoxical arousal, impaired consciousness, and passing into a coma. Indeed, even the possibility of a fatal outcome is not excluded [143]. Therefore, careful monitoring of the therapeutic efficacy and toxicity of benzodiazepines is necessary.

In recent years, a number of studies have been published indicating that the long-term use of benzodiazepines increases the risk of dementia by an average of 50%, especially in older people [144,145,146]. Whittington et al. showed that both the short-term and long-term use of midazolam leads to hyperphosphorylation of the *Tau* protein (microtubule-associated protein). As a result, this protein loses its ability to stabilize microtubules and aggregates in the cell forming pathomorphological structures (neurofibrillary tangles), thereby contributing to the development or exacerbation of dementia [144].

## 6. State of the Art

As mentioned earlier, macrophages and microglia are the main cellular reservoirs for persistent viral infection [61,72,73]. Recently, with the help of a highly sensitive in situ hybridization method that allows detecting and visualizing DNA expression within intact cells and tissues, HIV-1 was first detected in the brain tissue of HIV-positive patients who had an undetectable viral load [147]. At the same time, it was found that the viral DNA was detected exclusively in the macrophages/microglia of the brain [147]. Ko et al. also detected viral RNA in a small number of patients with suppressed viral replication, which indicated spontaneous reactivation of the virus and/or continued low virus replication [147]. These data once again confirmed that macrophages and microglia cells of the brain are the sources of persistent HIV infection. Moreover, recent studies have shown that another possible reservoir of HIV-1 may be pericytes, which are cells that play a key role in the maintenance of blood capillaries in the brain and are involved in regulating the permeability of the blood–brain barrier [148,149,150]. Previously, it was found that pericytes express CCR5 and CXCR4 co-receptors, which are necessary for the binding and penetration of HIV-1 into the cell [151]. In vitro experiments have shown that pericytes can support HIV-1 replication (extracellular production of the virus peaked 2–3 days after cell infection) [149]. In vivo studies in a mouse model also demonstrated that pericytes can be infected with HIV-1 [149,150]. Active transcription in vivo was confirmed by in situ PCR, showing that pericytes obtained from mouse brain microvessels were positive for the presence of HIV-1 gag and for spliced rev mRNA [149]. The presence of HIV-1 was also detected in pericytes obtained from human brain microvessels [148].

Delivery of antiretroviral drugs to the hidden viral reservoirs in therapeutic concentrations will help to suppress HIV replication. Thus, studies on the pharmacokinetic properties of the new-generation antiretroviral drug dolutegravir (integrase inhibitor) used in ART regimens over the past 7 years have shown that this drug penetrates the CNS well [152]. Letendre et al. found that the concentration of this drug in the cerebrospinal fluid was similar to its unbound concentration in blood plasma and exceeded, in vitro, the 50% inhibitory concentration for the wild-type HIV-1 strain [152]. This indicates that dolutegravir can reach therapeutic concentrations in the CNS [152]. Unfortunately, this drug had to be excluded from the treatment regimen, as it caused depression and paranoid ideation in some patients [153,154,155]. Recently, promising results were obtained by Gong et al., with their new elvitegravir nanoformulation (poloxamer-PLGA nanoformulation loaded with elvitegravir (integrase inhibitor)) [156,157]. Using an in vitro BBB model (co-cultured endothelial cells with astrocytes), Gong et al. showed that elvitegravir nanoformulation provided improved BBB penetration and increased the suppression of HIV replication in infected human-monocyte-derived macrophages and human-monocyte-derived microglia-like cells after crossing the BBB compared to the original elvitegravir [156,157]. Using NSG mouse (NOD scid gamma mouse) and HIV-1 encephalitic (HIVE) mouse models, Gong et al. demonstrated nearly twofold higher levels of elvitegravir nanoformulation in vivo in the mouse brain and a trend of decreasing HIV-1 viral load in the CNS of the HIV-1-infected mice [157].

## 7. Conclusions and Future Perspectives

The likelihood of developing mental disorders is significantly increased in people who are at increased risk of acquiring HIV (men who have sex with men, injection drug users, sex workers, and transgender people), [1] as well as in people living with HIV, due to their prolonged activation of immune responses and localization of the virus in different parts of the brain. Since it has been proven that viral replication in the CNS begins immediately after infection with HIV-1, it is likely that neuronal dysfunction occurs already during the asymptomatic period of HIV infection, causing subclinical cognitive impairment. The main targets of HIV-1 invasion are macrophages and microglia, which become cellular reservoirs for long-term viral persistence in the CNS, playing an essential role in the onset and development of HIV-associated mental disorders. Free viral particles, as well as viral proteins, disrupt metabolic processes in glial cells and reduce their ability to maintain a normal neuronal metabolism. Additional factors, such as combination antiretroviral therapies, can increase metabolic disturbances in the central nervous system of an HIV-infected person due to the neurotoxicity of some commonly used antiviral drugs and, thus, accelerate the development of the mental pathology. In addition, the competitive interaction of prescribed psychotropic and antiretroviral drugs resulting in mutual changes in their pharmacokinetic and pharmacodynamic properties does not exclude the occurrence or exacerbation of mental disorders, which should be taken into account by physicians in the treatment of PLWH.

Currently, it is becoming clear that despite the use of highly active antiretroviral therapy (ART), which effectively suppresses virus replication, the latent reservoirs of HIV-1 are the most complex barrier that prevents the complete elimination of the virus in the body of an HIV-infected patient. Therefore, it seems necessary to focus future research on studying the complete and safe (excluding the inflammatory nature of virus reactivation) eradication of HIV-1 in various latent reservoirs, especially in macrophages and microglia, which serve as a permanent source of the virus.

Another promising area of research, in our opinion, is the development of strategies aimed at the enhancement of antiretroviral drug permeability across the BBB. As already noted, many data have been accumulated, indicating that an overwhelming number of drugs cannot penetrate the CNS tissues [5,6,86]. It is assumed that the new generation of drugs should have the ability to penetrate the CNS without causing serious side effects. A drug-based nanocarrier is a highly promising delivery strategy to overcome the BBB and suppress HIV-1 viral replication in macrophages and microglia. In general, the development of various methods aimed at improving drug delivery to the CNS will be the subject of future research.

## Figures and Tables

**Table 1:** Pathological changes in CNS cells induced by HIV-1.

Cells	Co-Receptors Expressed on the Cell’s Surface	HIV-1 Induced Pathological Changes
Microglia Macrophages	CD4, CCR5, CXCR4 Full HIV-1 replication	Enhanced inflammatory processes via induced TNF-α and IL-6 production. Disruption of signaling pathways associated with apoptosis (via induced production of ROS and RNS). Cell cycle arrest, DNA and protein damage. Development of HIV-induced dementia.
Astrocytes	Very few express CCR5, CXCR4 Limited HIV-1 replication, do not produce full-ledged viral particles	Astrogliosis. Rapid death of neurons (via induced ROS production). Neuronal damage (via production of HIV-1 regulatory proteins *tat*, *nef*, *rev* by infected astrocytes). Development of HIV-1-associated neurocognitive disorders due to neuronal damage. Rapid development of dementia in patients without/low adherence treatment.
Oligodendrocytes	Limited expression of CXCR4 Debated if HIV-1 can replicate in oligodendrocytes in vivo	Incomplete cell’s maturation (via HIV-1 *tat* protein). Promotion of oligodendrocytes death (via HIV-1 *tat* protein). Dysregulation of myelin protein expression. Reduction oligodendrocytes’ ability to create myelin sheaths. Induction of apoptosis (via HIV-1 *tat* induced cells’ damage). Development of neurocognitive disorders.
Neurons	Do not express co-receptors Cannot be infected with HIV-1	Neuronal death due to the disruption of interneuronal connections induced by HIV-1 proteins (*tat*, *nef*, *gp120*). Changed morphology of neuronal phagosomes (via HIV-1 *tat* protein). Disruption of the autophagy process (via HIV-1 *nef* protein). Neurodegenerative disorders (via HIV-1 *nef* protein).

ROS—reactive oxygen species; RNS—reactive nitrogen species.

**Table 2:** Psychotropic and antiretroviral drugs interactions mediated by P450 metabolism.

Drug	P450 Cytochrome Substrate	Inhibitor	Inducer	Co-Administration of Drugs (Concentration)
**Antidepressants:**				
Fluoxetine	CYP2D6	CYP2D6, CYP3A4	-	PIs (ritonavir) ↑ NNRTIs (delavirdine) ↑
Paroxetine	CYP2D6	CYP2D6	-	PIs ↑
Sertraline	CYP2D6	CYP2D6	-	PIs ↑
Fluvoxamine	CYP2D6	CYP2D6, CYP1A2, CYP3A4	-	PIs ↑ NNRTIs (nevirapine) ↑
**Tryciclic Antidepressants (TCAs):**				
Trazodone	CYP3A4, CYP2D6	-	-	
Bupropion	CYP2B6	-	-	
Amitriptyline	CYP2D6	-	-	
Desipramine	CYP2D6	-	-	
Doxepin	CYP2D6	-	-	
Imipramine	CYP2D6, CYP1A2	-	-	
Nortriptyline	CYP2D6	-	-	
**Neuroleptics:**				
Risperidone	CYP3A4, CYP2D6	-	-	
Aripiprazole	CYP3A4, CYP2D6	-	-	
Quetiapine	CYP3A4	-	-	
Ziprasidone	CYP3A4	-	-	
Olanzapine	CYP1A2	-	-	
**Anxiolytics:**				
**Bensodiazepines**				
Alprazolam	CYP3A4	-	-	
Midazolam	CYP3A4, CYP2B6	CYP3A4	-	
Triazolam	CYP3A4	-	-	
Estrazolam	CYP3A4	-	-	
**Non-nucleoside reverse transcriptase inhibitors (NNRTIs):**				
Delaverdine	CYP3A4, CYP2D6	CYP3A4, CYP2B6	-	
Nevirapine	CYP3A4, CYP2D6	-	CYP3A4, CYP2B6	Fluoxetine ↓
Efavirenz	CYP3A4, CYP2B6, CYP2A6	CYP3A4, CYP2B6	CYP3A4, CYP2B6	Sertraline ↓ Bupropion ↑↓ (efavirenz dose dependent)
Etravirine	CYP3A4	-	CYP3A4	Midazolam ↑
**Protease inhibitors (PIs):**				
Ritonavir	CYP3A4, CYP2D6	CYP3A4, CYP2B6, CYP2D6	CYP3A4, CYP2B6, CYP1A2	Risperidone ↑ Aripiprazole ↑ Ziprasidone ↑ Quetiapine ↑ Trazadone ↑ Benzodiazepines ↑ ↓ Bupropion ↓ Paroxetine ↓ Sertraline ↓ Olanzapine
Darunavir	CYP3A4	CYP3A4	-	Risperidone ↑ Quetiapine ↑ Aripiprazole ↑ Trazadone ↑ ↓ Paroxetine ↓ Sertraline
Nelfinavir	CYP3A4, CYP2D6	CYP3A4, CYP2B6	CYP2B6	Bupropion ↑
Indinavir	CYP3A4	CYP3A4	-	Risperidone ↑ Aripiprazole ↑
Saquinavir	CYP3A	CYP3A4	-	Midazolam ↑

## Data Availability

Data sharing not applicable.
